# A review of the clinical utility of INR to monitor and guide administration of prothrombin complex concentrate to orally anticoagulated patients

**DOI:** 10.1186/1477-9560-10-5

**Published:** 2012-04-30

**Authors:** Sacha Sølbeck, Sisse R Ostrowski, Pär I Johansson

**Affiliations:** 1 Section for Transfusion Medicine, Capital Region Blood Bank, Rigshospitalet University of Copenhagen, Blegdamsvej 9, DK-2100, Copenhagen, Denmark

**Keywords:** INR, PCC, OAC, Anticoagulation reversal, Haemostatic efficacy

## Abstract

**Background and objectives:**

The number of patients treated with oral anticoagulation (OAC) is increasing and these patients are monitored by International Normalized Ratio (INR). Bleeding complications are common and we speculate if this is related to the limitation of INR only reflecting the initiation steps of the haemostatic process. The objective of the present review was to reassess the evidence for using INR as a tool to guide administration of prothrombin complex concentrates (PCC) to OAC patients. A Medline and Cochrane database search was conducted using the following keywords: prothrombin complex concentrate, reversal of oral anticoagulation and international normalized ratio (INR). Thirty-three articles were contracted and a total of ten studies were eligible after applying inclusion and exclusion criteria encompassing only 339 patients. No consensus regarding optimal target INR value to aim for when reversing OAC was found. In three of the studies it was reported that patients reaching their target INR continued to bleed, whereas three studies reviewed reported good haemostatic response also in patients that did not reach their target INR. The present review found limited evidence for the usefulness of INR as a tool to monitor and guide reversal of OAC induced coagulopathy in patients with PCC, which is expected given that it is a plasma-based assay only reflecting a limited part of the haemostatic process.

## Introduction

More and more people in the economically developed world are treated with oral anticoagulants due to the increasing proportion of elderly and the well documented effectiveness of anticoagulants to prevent primary and secondary thromboembolic events [1,2]. The most commonly used oral anticoagulants (OAC) are the synthetic coumarin derivatives warfarin, acenocoumarol and phenprocoumon, which are indicated in patients with atrial fibrillation and prosthetic cardiac valves [2].

Even though new oral anticoagulants have recently been introduced such as Dabigatran (FIIa inhibitor), and Apixaban (FXa inhibitor) which are being implemented as preventive drugs after knee and hip replacement surgery to avoid post operative complications [3], coumarin is still the drug must often chosen because it is cheap and well-documented. Synthetic coumarin derivates act by inhibiting synthesis of K-vitamin dependent coagulation factors II, VII, IX and X thereby inhibiting/reducing thrombin generation [4]. Treatment with coumarin derivates is associated with an increased incidence of bleeding, which is found to be a recognized complication affecting approximately 1% of the patients treated with OAC and about half of these patients die from bleeding complications annually [5]. Current treatment of choice for rapid reversal of patients on OAC in case of bleeding or need for acute surgery is prothrombin complex concentrate (PCC) encompassing coagulation factor II, VII, IX, X as well as Protein C and S [1]

Patients treated with OAC are routinely monitored by a coagulation analysis based on the prothrombin time (PT) that was introduced more than 50 years ago, when it was believed that the coagulation process occurred only in the plasma [6].

With the introduction of the cell-based model of haemostasis it became apparent that these assays only reflect the initiation step of the haemostatic process [7]. Therefore, whole blood viscoelastical assays (VHA), such as thrombelastography (TEG) or rotational thrombelastometry (ROTEM), that analyze the viscoelastic properties of clot formation in whole blood, including the patient’s ability to generate the thrombin, thereby reflecting the entire haemostatic process, these may be valuable in addition to plasma-based coagulation assays in OAC patients [8]. VHA has been reported to better predict transfusion requirements than PT and INR and are recommended by recent international guidelines for massively bleeding patients [9]. It should be noted, however, that no randomized clinical trial has been performed comparing the value of VHA vs. INR in predicting bleeding in OAC patients and consequently the value of VHA in this setting remains elusive.

The appropriateness of INR as a tool to monitor and guide OAC may be questioned by reviewing the literature concerning administration of PCC to reverse OAC treatment rapidly since coagulation factors II, VII, IX and X hereby is administered specifically and these are supposed to be reflected by this assay. The aim of the present review was, therefore, to provide an overview of the current literature reporting on the effect of administration of PCC to OAC patients prior to surgical intervention or in the event of bleeding and more specifically to review its effect on correction of INR as well as the association with achievement of haemostasis.

## Method

A literature search was conducted from PubMed and Cochrane Library with the aim of contracting articles reporting the use of PCC for reversal of OAC patients prior to surgical interventions or in the event of active bleeding, who were reporting the effect of the intervention on both INR and on clinical efficacy.

The search was conducted using the following key words: Prothrombin complex concentrate, reversal of oral anticoagulation, international normalized ratio. ("prothrombin"[MeSH Terms] OR "prothrombin"[All Fields]) AND complex[All Fields] AND concentrate[All Fields] AND Reversal[All Fields] AND ("mouth"[MeSH Terms] OR "mouth"[All Fields] OR "oral"[All Fields]) AND anticoagulation[All Fields] AND ("international normalised ratio"[All Fields] OR "international normalized ratio"[MeSH Terms] OR ("international"[All Fields] AND "normalized"[All Fields] AND "ratio"[All Fields]) OR "international normalized ratio"[All Fields]).

The following inclusion criteria were applied: 1. English language, 2. Human study, 3. Administration of PCC to reverse anticoagulation in bleeding patients or patients undergoing surgery, 4. Reporting end points including both effect on INR and clinical efficacy.

Exclusion criteria applied were: 1. In vitro studies and 2. Dental procedures.

## Results

Thirty-three articles were identified in the Medline search, whereas no articles were found in the Cochrane Database. After applying inclusion and exclusion criteria seven articles remained and another three studies were identified through the references of the identified reports totalling ten eligible studies (Table 1.)

**Table 1:** Description of the type of studies, number of patients included and patient group, dose of PCC, target INR, outcome 1 and 2, transfusions and thromboembolic events

**Author**	**Study**	**No.**	**Area**	**Intervention** **dose PCC/kg**	**Target INR**	**Outcome 1**	**Outcome 2**	**Transfusion + Vit. K**	**Thromboembolic events**
**8) Demeyere et al. 2010**	Randomised clinical trial	40 (20 pt. treated with PCC, 20 with FFP). 9 patients reached target INR spontaneously which leaves 16 pt. in PCC group	Cardiopulmonary	PCC (PPSB-SD Cofact, SANQUIN, CAF-DCF, Belgium, factor 4, inactive dosage based on pt weight, initial and target INR	<1.5	No. of patients reaching and time to target INR. 6/15 reached target INR after 60 min. (Data for one was missing and 4 had spontaneous normalization of their INR) 6/20 in PCC group needed additional PCC	Amount and no. of postoperative bleedings. None in PCC group suffered from post-op bleeding	33 U of red blood cells for 16 pt. in PCC group. No mention of vitamin K	None
**12) Van Aart et al. 2005**	Open, prospective, randomized controlled trial w. two arms	93	Invasive procedures or active bleeding	PCC (Cofact, Sanquin, Amsterdam, the Netherlands, factor 4). Treatment for group A: single dose of 20 ml PCC corresponding with 500 IU/ 7IU/kg. Group B: individualized dose based on pt weight, initial and target INR	Target INR for small intervention or minor bleeding: 2.1, 1.5 for major bleeding	After 15 min. 43% had reached target INR in group A respectively 89 % in group B	In group A three pt. continued to bleed and was given a 2nd dose PCC. In group B one pt. continued to bleed and was given a 2nd dose PCC. They had reached their target INR. All interventions were performed without bleeding complications and all existing bleeding stopped	No FFP or platelet concentrates were administered. No information on packed red blood cells. All pt received vitamin K	Two non-fatal non-bleeding cerebrovascular events occurred, one in each group
**13) Pabinger et al.** **2007**	Prospective multinational clinical trial	43	Intervention (26) or acute bleeding (17)	PCC (Beriplex P/N, CSL Behring GmbH, Marburg, Germany, factor 4) 25-35-50 IU/kg depending on initial INR 2-3.9, 4-6 or >6	<1.3 - 1.3	93% INR normalization to < 1.3 30 minutes after PCC. In the remaining three pt. INR was 1.4	Clinical haemostatic efficacy: 98% judged ”very good” or ”satisfactory”	Nothing on transfusions reported. 88% received vitamin K	One fatal pulmonary embolism
**14) Lorenz et al. 2007**	Prospective cohort study	8	Invasive procedures or active bleeding	Mean PCC (Beriplex P/N, CSL Behring GmbH, Marburg, Germany, factor 4) doses 57IU/kg in first round. 2 pt. received a second dose of 1500IU and 3500IU respectively	No target INR listed	Changes from baseline INR at 10, 30 and 60 min after PCC. Mean baseline INR 3.4 >10 min 7/8 INR <1.3, 1/8 INR:1.4	Clinical effectiveness rated by cessation of existing bleeding: 7/8 rated very good, 1/8 satisfactory	No transfusions reported. One pt received vitamin K	None
**15) Riess et al. 2006**	Open-labelled prospective clinical study	60	Major and minor surgery	PCC(Octaplex, Octapharma, Vienne, Austria, factor 4) 41.4 IU/kg PT% endpoint-PT% BL*kg = IU	Major surgery 1.3-1.1, Minor surgery 2.1-1.5	Target INR after PCC at 10, 30 and 60 min. 88%/91% respectively met criteria	Clinical efficacy, 3 point verbal scale (none, moderate, excellent) 56/56 was rated ”excellent” even though 5 did not respond in regard to INR	No transfusions reported. 24 pt received vitamin K	None
**16) Yasaka et al. 2004-05**	Prospective study	42	27 cerebral haemorrhage, 15 with major nonneurological haemorrhage or invasive procedures	PCC (PPSB-HT Nichiyaku, Nihon Pharmaceutical, Toyko, Japan, factor 4) 200-1500IU. INR after 10 and 60 min.	No target INR listed	30 pt. received median 8.8IU/kg, values decreased from median 2.49 to 1.19. 18.4IU/kg and 26.0IU/kg INR values decreased from median 2.33 to 0.96	Clinical evaluation: enlargement of haematoma in two pt. the one re-increased in INR from 1.48 to 2.72 half a day after INR reversal by 1000IU PCC, the other had syst. BP >200mm hg and low INR. 6 pt were evacuated with easy haemostasis during operation	No transfusions reported. 31/42 pt received vitamin K	None
**17) Lavenne-Pardonge et al. 2006**	Prospective study	14	Mixed group in immediate need of reversal	PPSB-Solvent Detergent by C.A.F.-D.C.F., factor 4) PCC dosed after weight. All pt. received only one dose	INR <2 for moderate bleeding, <1.5 for severe bleeding and cardiovascular procedure	11/12 reached target after 15 min and 13/14 after 60 min. (1/14 had an INR of 1.56)	Bleeding stopped in the 6 pt. treated for this/ no bleeding complications occurred during surgery for pt. treated prior to an operation	No transfusion reported. 80% received vitamin K	None
**9) Lubetsky et al. 2004**	Prospective non-randomized, non-controlled, open-labelled, multicenter phase II study	20	Bleeding (10) Surgery (10)	Octaplex (Octapharma, Vienna, Austria, factor 4): Bleeding group: 23.6IU/kg +/-7.0, surgery group: 31.2IU/kg +/-7.6	No target INR listed	Baseline INR in bleeding group: 7.1+/-2.5, surgery group; 5.0+/-2.8. Mean INR values declined 6.1+/-2.8 to 1.5+/-0.3 within 10 minutes.	Three point scale, good, moderate or none 1h post-infusion.Bleeding group 9/10 was rated good, 1/10 moderate. Surgery group: 8/10 good, 2/10 moderate. No information on the INR of these three pt.	All three were transfused with 2-7 units of packed cells. 7/20 vitamin K, median dose 2.3 mg	None
**18) Schick et al. 2009**	Retrospective study of case notes	50 (12 pt treated w. anticoagulant in need of reversal)	Intervention	(Beriplex P/N. CSL Behring, Marburg, Germany, factor 4) Median dose 1500 IU, 22IU/kg	<1.7	Change in baseline INR after PCC. Mean INR decreased significantly from baseline 2.8 to 1.5 at 180+/-31 min.	Bleeding: No major per operative bleeding was reported	One pt. was in need of blood component replacement therapy with FFP, platelets and red blood cells, after PCC administration7/12 received vitamin K	None
**19) Chong et al. 2009**	Observational study	7	Neurological 4 with subdural hematoma,3 had intracerebrale hematomas	PCC (Profilenine SD, Grisols Biologicale Inc, Los Angeles, CA, USA, factor 3), Median dose 28.5IU/kg	Normalization of INR defined as <1.5	Median time to INR normalization 420 min	Three died. Two had hematoma growth, one cause of dead not listed	6/7 pt received vitamin K	1 pulmonary embolism (in a pt. who had received a relatively lower dose)

### Randomised controlled studies (RCT)

Two RCT were identified. Demeyere et al. [10] compared the efficacy of intraoperative administration of PCC or FFP in 40 OAC patients undergoing heart surgery of whom 20 patients received PCC based on weight, initial and target INR (<1.5 or 1.5). None of the patients suffered from abnormal bleeding post-CPB surgery though only 6 had reached target INR after 60 minutes. A total of 33 RBC units were transfused to the PCC group. No thromboembolic events were reported.

Van Aart et al. [11] investigated in 93 OAC patients the efficacy of a standard dosage of PCC (Group A) as compared to an individualized dosage (Group B) on reaching target INR (minor bleeding or intervention: <2.1 or 2.1, major bleeding: <1.5 or 1.5) and bleeding.

Group A received a single PCC dose (7IU/kg) and 43% reached target INR after 15 minutes whereas 89% reached their target INR in group B. Four patients (3 in Group A and 1 in Group B) continued to bleed even though they had reached target INR. No FFP or platelet concentrates were administered, no information on transfusion of packed red blood cells. Two cerebral thrombotic events occurred, one in each group.

### Prospective observational studies

Pabinger et al. [12] evaluated the efficacy and safety of PCC (Beriplex P/N) in 43 patients who either were in need of intervention (N = 26) or demonstrated active bleeding (N = 17) at a dose of 25-35-50IU/kg depending on initial INR. In 93% of the patients INR was reduced to below the target INR of 1.3 and in 98% of the patients the clinical efficacy was rated ’very good’ or ’satisfactory’. One patient was judged ‘questionable’ because of persistent bladder bleeding. No information on transfusions reported. One fatal pulmonary embolism was reported which was judged to be possibly treatment related.

Lorenz et al. [13] evaluated the effectiveness and safety of PCC for emergency reversal of OAC in 8 patients undergoing invasive procedures or demonstrating active bleeding. Median Beriplex P/N dose was 57IU/kg and two patients needed a second dose of 1500IU and 3500IU, respectively, to reach target INR (no target value listed). Clinical effectiveness was rated by cessation of bleeding and 7/8 was rated ’very good’ and 1/8 as ’satisfactory’. No transfusions were administered and no thrombembolic events were reported.

Riess et al. [14] investigated 60 patients in need of reversal of OAC treatment before surgery. Octaplex was administered at a mean dose of 41IU/kg and 90% reached target INR being 1.1-1.3 for major surgery or invasive procedures and 1.5-2.1 for minor surgery or other invasive procedures. Clinical efficacy was rated ’excellent’ in all patients by the surgeon. No transfusions or thromboembolic events were reported.

Yasaka et al. [15] investigated the optimal PCC dose for acute OAC reversal in 27 patients with cerebral haemorrhage and in 15 patients with major haemorrhagic complications or patients who were to undergo invasive procedures without defining a target INR value. Thirty patients received 9IU/kg decreasing INR from 2.5 to 1.2. Another group of patients received 18IU- 26IU/kg and INR decreased from 2.3 to 1.0. In the patients who had cerebral haemorrhages the haematomas enlarged in two cases despite normalization of INR. Bleeding ceased in the non-neurological haemorrhage group after administration of PCC.

Lavenne-Pardonge et al. [16] investigated the use of PPSB-SD for reversal of OAC within 15 minutes in 14 patients, aiming for a target INR of <2.0 for moderate haemorrhage and abdominal surgery and <1.5 for severe haemorrhage and cardiovascular interventions. Data were missing on two patients and 11/12 of the remaining patients reached target INR and bleeding arrested in the 6 patients treated for ongoing bleeding. No transfusions or thromboembolic events were reported.

Lubetsky et al. [17] reported an open-labelled phase II-study were efficacy and safety of Octaplex for rapid reversal of OAC was evaluated without defining a target INR value for the intervention.

Twenty patients were enrolled (10 in a active bleeding group and 10 in a surgery group). Mean INR declined from 6.1 ± 2.8 to 1.5 ± 0.3.A three-point scale was used for evaluation of clinical efficacy and in the bleeding group 9/10 was rated ’good’ and 1/10 was rated ’moderate’. In the surgery group 8/10 was rated ’good’ and 2/10 ’moderate’. It was not specified which patients had normalized their INR. The three patients who were rated ’moderate’ were transfused with 2-7 units of packed cells. No thromboembolic events were reported.

### Retrospective studies

Schick et al. [18] reviewed 50 patients of whom 12 were OAC patients receiving Beriplex P/N. The median dose administered was 1500IU or 22IU/kg and target INR was less than 1.7. Mean INR decreased significantly from baseline INR 2.8 ± 0.2 to 1.5 ± 0.1.

Interestingly no major per operative bleeding was reported despite that one patient received RBC, FFP and platelet concentrate after PCC administration. No thromboembolic events were seen.

Chong et al. [19] investigated 7 neurological OAC patients with intracranial haemorrhage and receiving 3-factor PCC (Profilnine SD). Median dose of PCC was 28IU/kg and median time to normalization of INR (defined as INR less than 1.5) was 420 minutes. Three patients died, two because of haematoma growth despite PCC administration. One patient experienced a pulmonary embolism.

## Discussion

Double-blind randomized clinical trials evaluating the suitability of INR to guide PCC reversal in OAC treated patients are lacking and the result of the present review do not allow for any such conclusion either.

Unfortunately the search only identified 10 studies involving merely 339 patients where the effect of PCC on INR and haemostasis was investigated and half of the studies involved less than 30 patients each. The low number of patients investigated is somewhat surprising considering that in UK alone more than 1 million patients are on OAC [20] and that the number of OAC patients is steadily increasing [21].

Apart from the low number of patients investigated, it was difficult to assess the clinical utility of INR as a tool to monitor OAC patients due to considerable heterogeneity in the studies evaluated. Firstly, no consensus appeared to exist regarding what target INR value that is appropriate to aim for when reversing of OAC is considered since this varied from 1.1 to 2.1. Furthermore, considerable differences in PCC doses were used in the studies investigated and also different PCC products containing different levels of vitamin K dependent coagulation factors were administered making it impossible to delineate a possible relation between the units of coagulation factor activity administered and its effect on the INR. This is further corroborated by a recent American College of Chest Physicians Evidenced-based Clinical Guidelines not providing any guidance on optimal target INR or optimal dosage regarding reversal with PCC [22].

In regard to the association between INR and haemostasis this is obviously challenging given the methodological problem presented above and furthermore the evaluation of the haemostatic response to PCC resulting in a lowered INR was not standardized with some studies using transfusions and clinical judgement [10,18] as endpoints and others using only clinical judgement [11-17,19]. Despite this, however, we found that at least 10/339 (3%) patients continued to bleed despite reaching their target INR. Bleeding complications in relation to OAC treatment is the most common cause of medical emergency department visits in the US accounting for more than 15% of the 170.000 estimated visits each year [23]. Furthermore, Demeyere et al. [10] reported no abnormal bleeding in the 9 out of 16 patient’s not reaching target INR and undergoing cardiac surgery and Riess et al. [14] also found a good clinical response in 5 patients not reaching target INR and collectively they report that in the 22% of the patients not reaching target INR normal haemostasis was observed further indicating that this analytical method is associated with significant clinical limitations. This is in alignment with Segal et al. [24] who in a review reported that abnormal coagulation tests such as INR did not predict bleeding in patients undergoing invasive procedures. Hence, we do not infer that a cohort of OAC patients with a prolonged INR has increased bleeding risk as compared to OAC patients with a normal INR but instead point towards the ability of INR to predict bleeding risk for the individual OAC patient.

It is well established that conventional coagulation assays do not identify hypercoagulability [25] i.e. increased thrombotic risk and this is corroborated in the present review by the finding that four (1.8%) patients with a normal INR value developed thrombembolic events.

In recent years systematic use of whole blood viscoelastic assays such as TEG and ROTEM has improved transfusion therapy and outcome in surgical patients including trauma [25]. More than 25 studies including three randomized controlled studies all report that TEG/ROTEM better predict bleeding and need for transfusion than routine coagulation analysis and also that transfusion requirements and need for re-do surgery is lower in patients treated based on TEG/ROTEM [26]. The scientific rationale for this difference is that TEG/ROTEM assess haemostasis in whole blood including the platelets and thereby enable evaluation of all three phases of clot generation (initiation, amplification, propagation) as well as the physical characteristics of the formed clot [26] and we speculate whether monitoring OAC patients with these assays might be beneficial.

The present review has limitations such as only including studies where PCC was evaluated and where INR was used to monitor the degree of anticoagulation. Furthermore, restricting our search to English language reports may also have omitted relevant studies.

In conclusion the present review found limited evidence for the usefulness of INR alone as a tool to monitor and guide reversal of OAC patients with PCC, which is expected given that it is a plasma-based assay. We suggest INR being used in conjunction with whole blood viscoelastic assays and well-designed randomized controlled trials evaluating these whole blood viscoelastic assays compared to INR in this setting are warranted.

## Competing interests

The authors declare that they have no competing interests.

## Authors’ contributions

SS reviewed the literature, drafted the manuscript, SRO read and revised the manuscript, PJ designed the study and assisted in drafting the manuscript. All authors read and approved the final version manuscript.
